# Action Real-Time Strategy Gaming Experience Related to Enhanced Capacity of Visual Working Memory

**DOI:** 10.3389/fnhum.2020.00333

**Published:** 2020-08-31

**Authors:** Yutong Yao, Ruifang Cui, Yi Li, Lu Zeng, Jinliang Jiang, Nan Qiu, Li Dong, Diankun Gong, Guojian Yan, Weiyi Ma, Tiejun Liu

**Affiliations:** ^1^The Clinical Hospital of Chengdu Brain Science Institute, MOE Key Lab for Neuroinformation, University of Electronic Science and Technology of China, Chengdu, China; ^2^Center for Information in Medicine, School of Life Science and Technology, University of Electronic Science and Technology of China, Chengdu, China; ^3^Faculty of Natural Science, University of Stirling, Stirling, United Kingdom; ^4^School of Human Environmental Sciences, University of Arkansas, Fayetteville, AR, United States

**Keywords:** action real-time strategy gaming, action video gaming, change detection task, contralateral delay activity, visual working memory capacity

## Abstract

Action real-time strategy gaming (ARSG)—a major genre of action video gaming (AVG)—has both *action* and *strategy* elements. ARSG requires attention, visual working memory (VWM), sensorimotor skills, team cooperation, and strategy-making abilities, thus offering promising insights into the learning-induced plasticity. However, it is yet unknown whether the ARSG experience is related to the development of VWM capacity. Using both behavioral and event-related potential (ERP) measurements, this study tested whether ARSG experts had larger VWM capacity than non-experts in a change detection task. The behavioral results showed that ARSG experts had higher accuracy and larger VWM capacity than non-experts. In addition, the ERP results revealed that the difference wave of the contralateral delay activity (CDA) component (size 4–size 2) elicited by experts was significantly larger than that of non-experts, suggesting that the VWM capacity was higher in experts than in non-experts. Thus, the findings suggested that prolonged ARSG experience is correlative with the enhancement of VWM.

## Introduction

Action video gaming (AVG), as a main type of video gaming, is becoming an important entertainment medium worldwide. In an AVG session, players keep track of multiple complex visual stimuli simultaneously and respond to the stimuli under stringent time pressure (Green and Bavelier, 2003, 2012), which demands for various cognitive abilities, such as visual processing, attention, working memory, multi-target-tracking skills, and inhibitory control (Green and Bavelier, 2007, 2012; Spence and Feng, 2010). Bavelier and Green have proposed that AVG may be a potential, new training tool that can be used to induce cognitive and brain plasticity (Gentile and Gentile, 2008; Bavelier and Green, 2019) as AVG: (1) consistently keeps players in their zone of proximal development through the appropriate selection of entry levels and gradual step-size change in difficulty; (2) constantly exposes players to a perceptually rich and cognitively challenging environment; and (3) motivates players to persist through challenging tasks using rewards and punishments. Thus, research should examine the possibility of using AVG as a new, computerized intervention approach to improve various aspects of cognition ability.

Recently, action real-time strategy gaming (ARSG)—a new genre of AVG that contains both *action* and *strategy* components—has grown in popularity. In addition to requiring sensorimotor skills [e.g., attention, visual working memory (VWM), visuo-spatial cognition, hand–eye coordination], ARSG also requires timely strategic decision-making and teamwork, just like traditional team sports (e.g., soccer, basketball). Thus, ARSG is a high-cognitive-load task, further offering promising insights into learning-related cognitive and neural plasticity. Using event-related potential (ERP) measurement, this study explores the effect of ARSG experience on cognitive and neural plasticity by determining whether ARSG experts have larger VWM capacity than non-experts.

This study examined the development of VWM because it is an important component of cognitive and brain plasticity. In addition, VWM is a critical cognitive mechanism for successful video gaming as it enables players to keep task-related visual stimuli on a short delay after its presentation (Logie, 2011; Blacker and Curby, 2013). Importantly, the VWM capacity is limited in humans (Luck and Vogel, 1997, 2013; Cowan, 2001; Hao et al., 2018) and varies across individuals (Cowan et al., 2005; Vogel et al., 2005; Cusack et al., 2009; Astle and Scerif, 2011), suggesting that the VWM capacity is susceptible to improvement according to one’s experience. Indeed past behavioral studies showed that long-term AVG experience was related to VWM improvement using a change detection task (Boot et al., 2008; Clark et al., 2011; Blacker and Curby, 2013; Wilms et al., 2013; Blacker et al., 2014; Li et al., 2015) and alternative tasks (Green and Bavelier, 2003, 2006; Sungur and Boduroglu, 2012; Colzato et al., 2013; Oei and Patterson, 2013; McDermott et al., 2014; Waris et al., 2019). Additionally, functional magnetic resonance imaging (fMRI) research showed that AVG experience was related to superior VWM ability and modulation in the activity of the fronto-parietal cortex dependently on task difficulty (Moisala et al., 2017). These findings suggested that the AVG experience may facilitate the development of VWM.

However, it is still unclear whether the ARSG experience is related to the development of VWM. Arguably, ARSG may benefit cognitive development just like AVG because both ARSG and AVG have the “action” component (Dale and Green, 2017a,b; Bavelier and Green, 2019; Dale et al., 2019). In addition, an fMRI study suggested that, compared with ARSG non-experts, experts had superior functional integration between salience and central executive networks which represent the attention and working memory severally (Gong et al., 2016). A recent EEG study demonstrated that the EEG theta-band power (related to working memory load) was stronger during ARSG playing than during resting, music listening, and non-AVG gaming (i.e., life simulation gaming; Gong et al., 2019a). Furthermore, ERP research showed that prolonged ARSG experience (e.g., League of Legends) was related to enhancement of visual attention, which was closely associated with individual differences in the VWM capacity (Qiu et al., 2018; Gan et al., 2020). Thus, ARSG offers a new perspective on cognitive and neural plasticity (Gong et al., 2015, 2017; Kowalczyk et al., 2018; Gong et al., 2019a,b).

The present study examined the relationship between ARSG experience and VWM capacity by comparing ARSG experts’ and non-experts’ behavioral and ERP data in a change detection task—a test typically used to estimate the VWM capacity. The originality of this study lies in the game genre, the measure technology, and the cognitive task used. First, unlike previous studies using AVG genre (Boot et al., 2008; Blacker and Curby, 2013; Oei and Patterson, 2013; Blacker et al., 2014; Waris et al., 2019), this study utilized ARSG—an increasingly popular genre in recent years. Second, this study used ERP measures to test time-sensitive indicators to sub-processes underlying VWM (Luck, 2014), which may not be readily observable in behavioral research (Dale and Green, 2017a; Dale et al., 2019). Third, the participants performed a change detection task that is indicative of VWM capacity rather than attention tasks (Qiu et al., 2018; Gan et al., 2020). Although VWM is closely related to several aspects of attention (Vogel et al., 2005; Astle and Scerif, 2011), visual attention and VWM share different key capacity-limited mechanisms (Howard et al., 2020). Thus, it is still unknown whether ARSG experience is related to an enhanced VWM capacity.

We would predict that ARSG experts have a higher accuracy rate and larger VWM capacity than non-experts based on previous behavioral findings (Blacker and Curby, 2013; Oei and Patterson, 2013; Blacker et al., 2014). Furthermore, the between-groups differences in the VWM capacity should be indicated by the differential wave of contralateral delay activity (CDA) between a four-item array and a two-item array (size 4–size 2; Vogel et al., 2005). The CDA component is a negative slow-wave component whose amplitudes get largest during 300–900-ms time window after memory array beginning over posterior the region. The CDA amplitude increased as the number of objects maintained in WM increased; its amplitude gets an asymptotical limit based on each individual’s memory capacity (Vogel and Machizawa, 2004; Vogel et al., 2005; Luria et al., 2016; Zhang et al., 2020). The VWM capacity is indicated by the difference wave of CDA component between a four-item array and a two-item array; thus, this difference is greater in high-capacity individuals than in low-capacity individuals. Thus, we would predict that ARSG experts should elicit a larger difference wave of CDA component (size 4–size 2) than non-experts.

## Materials and Methods

### Participants

This study used the recruitment procedure established in previous research (Qiu et al., 2018; Gong et al., 2019b; Gan et al., 2020). All the participants in the current study were college students of the University of Electronic Science and Technology of China (UESTC) who responded to the recruitment flyers posted on campus or on the Internet forums hosted by the UESTC. Prior to this experiment, the participants completed a questionnaire that collected demographic information, including age, sex, color vision, handedness, and history of mental and neurological diseases. None of them reported having a history of mental and neurological diseases. The participants also completed a Self-Rating Depression Scale (SDS), a Self-Rating Anxiety Scale (SAS), and a seven-question Game Addiction Questionnaire. Based on the results, eight additional individuals were excluded prior to the experiment because they had moderate/severe depression or anxiety (i.e., SDS ≥ 63 or SAS ≥ 60; *n* = 5) and Internet gaming disorders (*n* = 3). The participants also reported their: (1) gaming experience in the recent 2 years and expertise gaming ranking of League of Legends (LOL)—the ARSG game used in this study; (2) LOL ID, which was used to validate the participants’ self-reported gaming experience and expertise; and (3) gaming experience of other gaming genres in the recent 2 years to ensure that LOL was the major game genre for all the participants (Qiu et al., 2018; Gong et al., 2019b; Gan et al., 2020). All the participants were right-handed and had normal or corrected-to-normal vision and no history of mental and neurological diseases.

This study defined the experts and the non-experts according to both time- and skill-based criteria established in previous studies (Qiu et al., 2018; Gong et al., 2019b; Gan et al., 2020). The experts had at least 2 years of LOL gaming experience and were LOL masters based on their expertise gaming ranking (the top 7% of players)—an objective, commonly used tool for quantitating the relative gaming skill levels of LOL players. The non-experts had less than 0.5 years of LOL gaming experience and were non-experts according to their expertise gaming rankings (the lowest 29.92–45.11% of players; Qiu et al., 2018; Gan et al., 2020). To minimize participant bias, the participants were not notified of their group membership or the aim of this experiment. The sample size was determined by G*Power 3.1.9 software, which gave the effect size of 0.25, the power of 0.80, and the alpha of 0.05 (Faul et al., 2007). Forty-one males who were healthy undergraduate and graduate students of UESTC were recruited. Four participants were excluded from the final sample because their accuracy rates in the tasks were below chance (i.e., 50%) and the EEG data had noise of more than 25%. In total, 18 LOL experts (*M* = 20.79, SD = 3.90; *n* = 18) and 19 non-experts (*M* = 20.06, SD = 1.30; *n* = 19) were recruited.

### Fluid Intelligence Measurement

Since fluid intelligence is correlated with the VWM capacity (Cowan et al., 2005; Cusack et al., 2009; Fukuda et al., 2010), we collected the participants’ fluid intelligence data as in Blacker et al. (2014). Prior to the experiment, the participants were administered a Ravens Progressive Matrices test (RPM, a standardized non-verbal measure of fluid intelligence), which is normalized in China (Raven et al., 1990). The reliability and the validity of the RPM version were from 0.79 to 0.95 and from 0.54 to 0.71, respectively (Zhang and Wang, 1989). This test presented a complex visual pattern that a piece was cut out, and it required the participants to get the absent piece to complete the pattern. The entire RPM included 60 items, and the participants were given 45 min to complete the test. Here we reported the original scores that were not converted into standard IQ scores.

### Apparatus and Stimuli

Using E-prime software to present stimuli on a monitor (refresh rate, 60 Hz; model, L2250pwD, 22 inches; height, 30 cm; width, 47 cm). The participants were seated 60 cm in front of a monitor in a recording room with electrical and sound shielding. The memory items consisted of a series of colored squares, the colors of which were randomly selected without replacement from eight easily recognizable colors (RGB values, red: 255, 0, 0; cyan: 0, 255, 255; blue: 0, 0, 255; black: 0, 0, 0; yellow: 255, 255, 0; white: 255, 255, 255; violet: 238, 130, 238; green: 0, 255, 0). The visual angle of every colored square was 0.65° × 0.65° (Vogel and Machizawa, 2004; Vogel et al., 2005; Hao et al., 2018).

### Procedure

**Figure 1:**
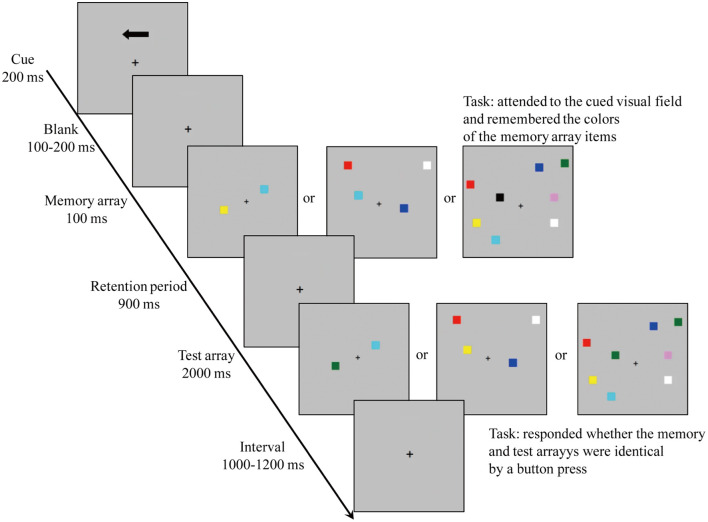
The experimental procedure.

To measure the VWM capacity, the participants were administered a visual change detection task (see Figure 1; Vogel and Machizawa, 2004; Vogel et al., 2005; Hao et al., 2018). The items were presented within 4° × 7.3° rectangular regions bilaterally and centered 3° to the left and the right of the middle of the monitor. The memory arrays included one, two, or four items which were chosen randomly in each hemifield, and the colors of the items did not repeat in the same hemifield. The stimuli locations were randomized, and the distance between items in each hemifield was at least 2°.

First, a fixation point (“+”) was presented in the middle of the gray background (RGB: 192, 192, 192). Then, one arrow cue was presented above fixation (200 ms), indicating which side would be followed on this trial. After a random interval (100–200 ms), a memory array appeared (300 ms). Following a retention period (900 ms), the test array was presented and then disappeared after either 2,500 ms or completing a response. After a random interval (1,000–1,200 ms), the next trial was started. The participants were informed to hold their eyes on the center of screen while storing the colors of the items in the hemifield pointed through the arrow cue. The participants were asked to decide whether the color of the test array was different than that of the memory array by pressing a key on the keyboard interfaced to a computer (identical: press 1; different: press 2). Accuracy, rather than speed, was stressed to ensure that any between-groups performance differences reflected the VWM-related information rather than the speed-related information that has been shown as improvable by video gaming experience (Blacker and Curby, 2013; Blacker et al., 2014; Dale and Green, 2017b).

The colors of the test arrays in the cued hemifield were identical to the colors of the memory arrays in half of the trials (i.e., “un-change” trials), while they were different in the remaining trials (i.e., “change” trials). During “change” trials, an unused colored item in the memory hemifield was randomly chosen from the rest of colors. Meanwhile, 50% “change” trials were presented in the left of fixation, the remaining “change” trials were presented in the right of fixation. There were three levels of size set (one, two, and four sizes); therefore, there were 12 conditions in all intermixed within blocks [size (1 vs. 2 vs. 4) × color (change vs. un-change) × location (left vs. right)] for each group. All the participants performed at least 24 trials to ensure that they know the experimental instructions; there were a total of 12 blocks with 60 trials in each block, resulting in a total of 720 trials.

### EEG Acquisition and Preprocessing

The behavioral and the EEG data were recorded simultaneously while the participants performed the visual change detection task. The EEG signals were recorded by an EEG32-BT EEG amplifier (BORUIEN, China), the electrodes were located according to the 10-20 system, and the signals were digitized with a 1,000-Hz sampling rate (Klem et al., 1999). All signals were online-filtered with a 0.05–100-Hz bandpass filter and were online-referenced to the FCz. The impedance for all electrodes was kept below 5 KΩ.

The offline EEG analyses were conducted by EEGLAB (Delorme and Makeig, 2004) and ERPLAB toolboxes (Lopez-Calderon and Luck, 2014) in MATLAB 2013b (MathWorks, Natick, NA, USA). The EEG data were first re-referenced to “infinity” zero operated by the reference electrode standardization technique (Yao, 2001; Dong et al., 2017). The re-referenced data were then filtered by using IIR-Butterworth non-causal filters, with half-power cutoffs of 0.10 and 30 Hz (roll-off = 12 dB/oct), and two-order 49–51-Hz notch filter was also used to eliminate power frequency interference. The filtered data were segmented into 1,100-ms epochs which were time-locked to the memory array beginning and included a 100-ms pre-stimuli baseline. Noisy trials were deleted by the moving window peak-to-peak amplitude method, with 200-ms window width, 100-ms window step, and 100-μV threshold, and the simple voltage threshold method with 100-μV threshold. To reduce the interference of saccadic eye movements and blinks, the step method was used with 400-ms window width, 200-ms window step, and 15-μV threshold (Luck, 2014). Data of participants whose 25% of trials were defined as noise were deleted from further analyses. Three participants were excluded based on this criterion. Among the responses to “un-change” trials, press “1” responses were “correct” responses; among the responses to “change” trials, press “2” responses were “correct” responses. Only the trials of “correct” responses were included in the next analysis. We averaged the single-trial signals to get the individual-level amplitudes and averaged the individual-level amplitudes to get the group-level average amplitudes. We smoothed the group-level average amplitudes by a 20-Hz low-pass filter for plotting (Luck, 2014).

### Data Analysis

To test the difference in fluid intelligence between experts and non-experts, we conducted independent-sample *t*-test for the RPM original scores of experts and non-experts. For accuracy, 2 (group: experts vs. non-experts) × 3 (size: 1 vs. 2 vs. 4) two-way analysis of variance (ANOVA) was conducted. The “change,” “un-change,” left, and right trials were collapsed and averaged to ensure statistical power. To further compare the VWM capacity between expert and non-expert groups, two-sample *t-test* was conducted for the *K*-values of experts and non-experts. Specifically, the hit rates (ratio of identified “change” trials correctly) and the correct rejection rates (ratio of identified “un-change” trials correctly) were first calculated respectively for each size. *K* was then calculated [*K* = set size × (hit rate − false alarm rate)] as a marker of the VWM capacity (Vogel and Machizawa, 2004; Cowan et al., 2005; Vogel et al., 2005). *K*-values of different sizes were averaged to obtain a single and robust measure of the VWM capacity within each participant (Weaver et al., 2017; Feldmann-Wustefeld and Vogel, 2019).

For EEG data, we focus on the CDA component. Since previous studies showed that the CDA amplitude is the most evident around the posterior regions, including the posterior parietal, lateral occipital, and posterior temporal electrode sites (Vogel and Machizawa, 2004; Vogel et al., 2005), five electrode pairs at the posterior regions (CP1/CP2, CP5/CP6, P3/P4, P7/P8, and O1/O2) were selected for further analyses. The contralateral amplitude was the averaged ERP activity of the left hemisphere electrode sites while cueing the participants to attend the memory array of the right hemifield and vice versa. Similarly, the ipsilateral amplitude was the averaged ERP activity of the left hemisphere electrode sites while cueing the participants to attend the memory array of the left hemifield and vice versa. Then, the CDA component was computed by subtracting the ipsilateral amplitude from the contralateral amplitude and averaging the CDA amplitude during 300–900-ms time window after the memory array beginning (Vogel and Machizawa, 2004; Vogel et al., 2005; Luria and Vogel, 2011; Luria et al., 2016). The “un-change” and “change” trials were collapsed and averaged, and the five electrode pairs were collapsed and averaged to increase the statistical power. Based on previous studies (Vogel et al., 2005), 2 (group) × 3 (size) two-way ANOVA cannot be conducted. To test the difference of CDA amplitude among the three sizes for each group, one-way ANOVAs were conducted for each group, respectively. To further compare the VWM capacity of experts with that of non-experts, independent-sample *t*-test was conducted to compare the difference wave of CDA (size 4–size 2) of experts with that of non-experts. Bonferroni correction was used for multiple comparisons. For all analyses, the significance level was specified as 0.05. When the Bonferroni correction was used for multiple comparisons, the significance level was 0.05 divided by the number of statistics.

## Results

### Fluid Intelligence

First, a square transformation was conducted to transform the data to ensure that the distribution of scores matched the assumptions of the *t*-test. To test the difference in fluid intelligence between both groups, an independent-sample *t-test* then compared the original RPM scores between the experts and the non-experts. The results indicated that the fluid intelligence scores did not differ between the experts (*M* = 57.83, SD = 2.01) and the non-experts (*M* = 57.11, SD = 2.11; *t*_(35)_ = 1.16, *p* = 0.26).

### Behavioral Results

**Figure 2:**
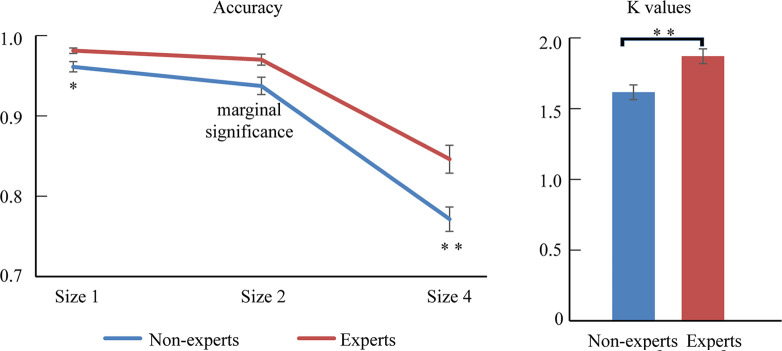
The difference between experts and non-experts in accuracy and *k*-values. Error bars represent mean ± SEM in the left, after correction by Bonferroni method. *p < 0.017, **p < 0.003; in the right, **p < 0.01.

The accuracy was mostly at ceiling level; thus, an arc sine transformation was conducted to transform the accuracy to ensure that the distribution of data matched the assumptions of the ANOVA tests. Then, 2 (group: experts vs. non-experts) × 3 (size: 1 vs. 2 vs. 4) two-way ANOVA analyzed the accuracy data (see Figure 2 and Table 1). The results revealed that the main effect of size was significant, with accuracy being higher for smaller sizes (*F*_(2,70)_ = 246.06, *p* < 0.001, $\eta_{\text{p}}^{2}$ = 0.88); the main effect of group was also significant, showing that the experts had a superior overall accuracy over the non-experts (*F*_(1,35)_ = 13.76, *p* < 0.001, $\eta_{\text{p}}^{2}$ = 0.28). However, the size × group interaction did not reach significance (*F*_(2,70)_ = 0.82, *p* = 0.44, $\eta_{\text{p}}^{2}$ = 0.02).

**Table 1:** Descriptive behavioral results in both experts and non-experts.

	Size 1	Size 2	Size 4	*k*
Non-experts	0.96 (0.03)	0.93 (0.05)	0.77 (0.07)	1.62 (0.22)
Experts	0.98 (0.01)	0.97 (0.03)	0.85 (0.07)	1.87 (0.22)

*The values are the mean (standard deviation) values in accuracy and *K*-values*.

Because this study focused on the differences between LOL non-experts and experts, we decomposed this interaction further. One-sample repeated-measures ANOVAs were conducted in each group, respectively. For the non-experts, the accuracy rate significantly differed across the three sizes (*F*_(2,36)_ = 164.04, *p* < 0.001, $\eta_{\text{p}}^{2}$ = 0.90), with the highest accuracy at size 1 and the lowest accuracy at size 4 (corrected by Bonferroni method). For the experts, the accuracy significantly differed across the three sizes (*F*_(2,34)_ = 93.66, *p* < 0.001, $\eta_{\text{p}}^{2}$ = 0.85), showing that the accuracy rates were significantly higher with size 1 and size 2 than with size 4; however, the accuracy rates did not differ between size 1 and size 2 (corrected by Bonferroni method). In addition, separate independent-sample *t*-tests were conducted for each size. The results showed that, compared with the non-experts, the experts had a significantly higher accuracy at size 1 (*t*_(35)_ = −2.92, *p* = 0.006, Cohen’ *d* = −0.96), size 2 (*t*_(35)_ = −2.88, *p* = 0.007, Cohen’ *d* = −0.95), and size 4 (*t*_(35)_ = −3.32, *p* = 0.002, Cohen’ *d* = −1.09; corrected by Bonferroni method).

We then conducted an independent-sample *t*-test to compare the mean VWM capacity [i.e., *K* = set size × (hit rate − false alarm rate)] between the experts and the non-experts (see Figure 2 and Table 1). The mean VWM capacity was 1.62 items in the non-experts and 1.87 items in the experts. The results revealed that the experts had significantly larger *K*-values than the non-experts, suggesting that the experts had a larger mean VWM capacity than the non-experts (*t*_(35)_ = −3.49, *p* = 0.001, Cohen’ *d* = −1.15).

### ERP Results

**Figure 3:**
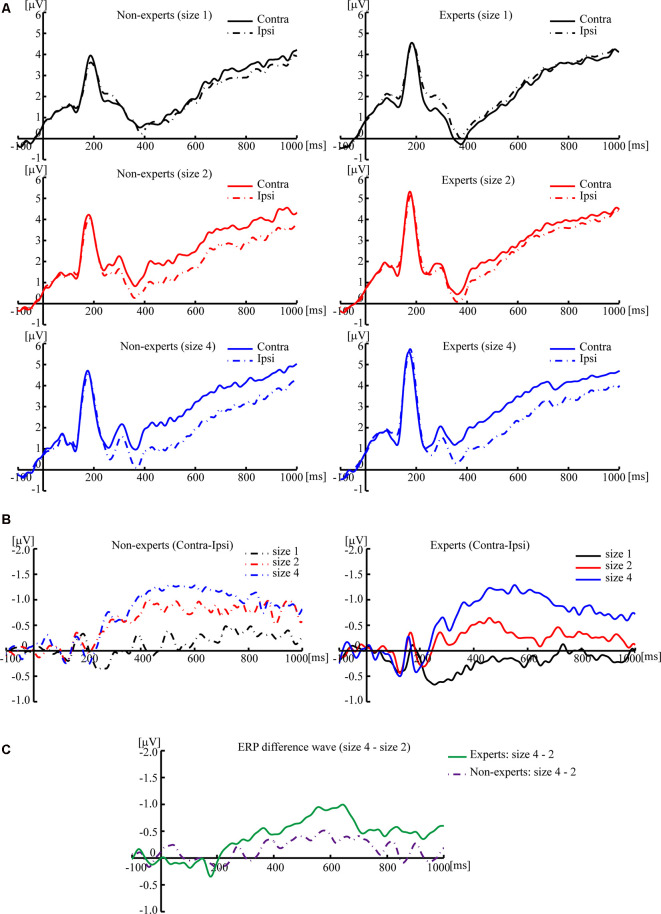
Grand averaged event-related potentials (ERPs), elicited by memory array onset with the League of Legends (LOL) non-expert and expert groups. **(A)** Grand averaged ERPs of ipsilateral and contralateral brain regions, elicited by memory array onset with the LOL non-expert (left panels) and expert groups (right panels) at size 1, size 2, and size 4, respectively. **(B)** Grand averaged ERP difference waves that subtracted the ipsilateral activity from the contralateral activity, elicited by memory array onset with the LOL non-expert (left panels) and expert groups (right panels) at size 1, size 2, and size 4, respectively. **(C)** Grand averaged ERP difference waves with the amplitude of size 4 minus the amplitude of size 2, elicited by memory array onset with LOL non-experts and experts. The grand averaged wave was time-locked to the memory array onset and averaged across the posterior electrode pairs (CP1/CP2, CP5/CP6, P3/P4, P7/P8, and O1/O2). By convention, a negative voltage is plotted upwards.

**Figure 4:**
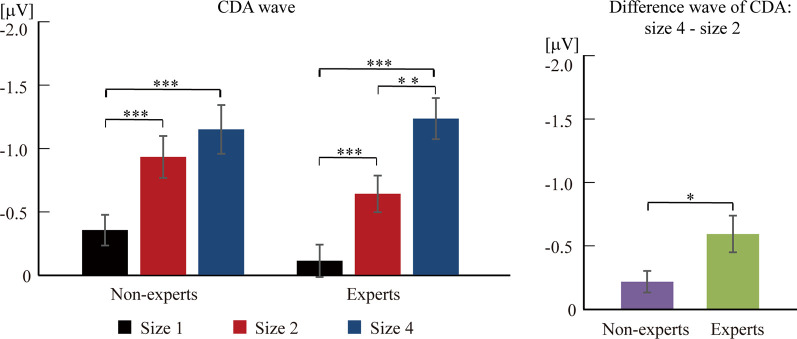
The difference wave of contralateral delay activity (CDA) component between experts and non-experts. The CDA wave was time-locked to the memory array and averaged across the posterior electrode pairs (i.e., CP1/CP2, CP5/CP6, P3/P4, P7/P8, and O1/O2) and across 300–900 ms after the memory array onset. Error bars represent mean ± SEM in the left, after correction by Bonferroni method. *p < 0.017, **p < 0.003, ***p < 0.0003; in the right, *p < 0.05.

Based on previous studies (Vogel et al., 2005), 2 (group) × 3 (size) two-way ANOVA cannot be conducted. Separate one-way ANOVAs were instead done to test the difference of CDA amplitude among three sizes within each group (see Figures 3, 4). For the non-experts, the CDA amplitude elicited across the three sizes was significantly different (*F*_(2,36)_ = 27.42, *p* < 0.001, $\eta_{\text{p}}^{2}$ = 0.60), showing that the CDA amplitudes elicited by size 2 and size 4 were significantly more negative than size 1, but the CDA amplitudes elicited by size 2 and size 4 did not differ significantly (corrected by Bonferroni method). For the experts, the CDA amplitude elicited across the three sizes reached a significant difference (*F*_(2,34)_ = 39.43, *p* < 0.001, $\eta_{\text{p}}^{2}$ = 0.70), with the most negative CDA amplitude at size 4 and the fewest negative CDA amplitude at size 1 (corrected by Bonferroni method). Notably, the CDA amplitude elicited by size 4 was more negative than that of size 2 within the experts.

Then, an independent-sample *t*-test was conducted to compare the difference wave of CDA (size 4–size 2) between the experts and the non-experts (see Figures 3, 4). The results showed that the difference wave of the CDA (size 4–size 2) elicited by the experts (*M* = −0.59, SD = 0.61) was significantly larger than that of the non-experts (*M* = −0.22, SD = 0.37), revealing that the experts had a higher VWM capacity than the non-experts (*t*_(35)_ = 2.24, *p* = 0.03, Cohen’s *d* = 0.74).

## Discussions

Using both behavioral and ERP measures, this experiment tested the relationship between the ARSG experience and the improvement of the VWM capacity—a critical component for inducing cognitive and neural plasticity. Both ARSG experts and non-experts completed a change detection task that is the typical experimental program used to examine the VWM capacity; meanwhile, the behavioral and the EER responses were measured. The behavioral and the ERP results suggested that the ARSG experts had a larger VWM capacity than the non-experts, suggesting that a long-term ARSG experience is related to an improved VWM capacity.

### ARSG Experts Outperformed the Non-experts According to the Accuracy Data

The ARSG experts outperformed the non-experts, as indicated by the finding that the accuracy was higher in the experts than in the non-experts at size 1, size 2, and size 4. Previous studies found that AVG experience is related to an improved VWM performance in several tasks (Green and Bavelier, 2003, 2006; Boot et al., 2008; Cusack et al., 2009; Clark et al., 2011; Sungur and Boduroglu, 2012; Blacker and Curby, 2013; Colzato et al., 2013; Oei and Patterson, 2013; Wilms et al., 2013; Blacker et al., 2014; McDermott et al., 2014; Li et al., 2015; Moisala et al., 2017; Waris et al., 2019). For example, Blacker et al. found that AVG experts had a VWM advantage in accuracy over non-experts in a change detection task, similar to our simple colored stimuli (Blacker and Curby, 2013; Blacker et al., 2014). Furthermore, given the hypothesis that the AVG experience enhances cognitive and brain plasticity because of its “action” mechanics, as AVG: (a) requires players to respond to stimuli under stringent time pressure; (b) puts a persistent, large cognitive demand on divided attention; and (c) demands for timely shifts between focused and divided attentions (Bavelier and Green, 2019), researchers have examined ARSG that integrates the “action” mechanics into real-time strategy gaming. Research demonstrated that the ARSG experience benefited various cognitive abilities just as the AVG experience (Gong et al., 2015, 2016, 2017; Dale and Green, 2017a,b; Kowalczyk et al., 2018; Qiu et al., 2018; Bavelier and Green, 2019; Dale et al., 2019; Gong et al., 2019a,b; Gan et al., 2020). Consistent with these findings, the current study found that the ARSG experience was related to the enhancement of the VWM capacity.

### ARSG Experts Have a Higher VWM Capacity Than Non-experts According to the *K*-Values

The ARSG experts have a larger VWM capacity than the non-experts as indexed by the experts’ larger *K*-values than those of the non-experts. The *K*-values have been used as a behavioral indicator of the VWM capacity in previous studies—a larger *K*-value indicates a higher VWM capacity (Cowan, 2001; Vogel and Machizawa, 2004; Vogel et al., 2005; Luria et al., 2016; Weaver et al., 2017; Feldmann-Wustefeld and Vogel, 2019). Moreover, several studies used the *K*-values to examine the influence of AVG experience on VWM capacity (Boot et al., 2008; Blacker and Curby, 2013; Blacker et al., 2014) and found that, compared to AVG non-experts, experts had larger *K*-values and that the *K*-values increased after AVG training. Thus, the current study supports the positive relationship between ARSG experience and the enhancement of the VWM capacity.

### ARSG Experts Have a Higher VWM Capacity Than Non-experts According to the Electrophysiological Marker—CDA Component

The VWM capacity reached an asymptotic limit faster within ARSG non-experts than experts as indicated by the finding that the CDA amplitudes elicited by size 2 and size 4 were not significantly different within the non-experts, but the CDA amplitude elicited by size 4 was more negative than that by size 2 within the experts. CDA can indicate the VWM capacity as CDA amplitude increased as the number of objects held in VWM increased, and its amplitude gets asymptotical limit for arrays that meet or exceed the capacity. High-capacity individuals tend to elicit a greater increase of CDA amplitude when larger sizes are maintained in VWM, supporting the proposition that high-capacity individuals have the enhanced abilities to store and process information (Luck and Vogel, 1997, 2013; Cowan, 2001; Vogel and Machizawa, 2004; Vogel et al., 2005; McCollough et al., 2007; Luria et al., 2016). Furthermore, CDA amplitude may reflect the currently active representations maintained in VWM rather than other task-relevant factors, such as the amount of general effort, the degree of executive control and the difficulty involved in the task, the number of spatial positions in VWM, and the stimuli contrast (Vogel and Machizawa, 2004; Ikkai et al., 2010; Luria et al., 2010, 2016; Gao et al., 2013; Ye et al., 2014). Thus, the CDA component was a reliable indicator of the VWM capacity, extending the previous behavioral findings that a long-term AVG experience was positively related to an enhanced VWM performance.

ARSG experts had a higher VWM capacity than non-experts, as indicated by the difference wave of CDA (size 4–size 2) that was larger in the experts than in the non-experts. Vogel et al. (2005) suggested that the VWM capacity limit can be estimated by a difference wave of CDA amplitude between a four-item array and a two-item array. Specifically, high-capacity individuals may elicit a greater difference wave of CDA amplitude than low-capacity individuals, suggesting that a two-item array consumed less available VWM capacity within high-capacity individuals than within low-capacity individuals. Therefore, the present findings revealed that the ARSG experts have a larger VWM capacity than non-experts, suggesting that the prolonged ARSG experience is related to enhancing the VWM capacity.

Moreover, individual differences in VWM capacity are related to general cognitive abilities. For example, such differences are related to the filtering efficiency of irrelevant visual information, and individual differences of CDA amplitude reflect both the ability to retain a different number of objects and the attentional control ability to select and protect task-relevant items and to filter irrelevant items (Vogel et al., 2005; Luria et al., 2016). Green and Bavelier (2012) suggested that enhanced selective attention supports the AVG players’ superior VWM (Green and Bavelier, 2012). In addition, the VWM capacity plays an important role in the transfer from working memory to target template (Schmidt et al., 2014). Furthermore, the VWM capacity is correlated with broad cognitive abilities, accounting for 46% of individual differences in expansive cognitive abilities (e.g., attention, reasoning, and social cognition) and 43% of individual differences in fluid intelligence (Fukuda et al., 2010; Luria et al., 2016). However, our result did not show a significant difference in fluid intelligence between ARSG experts and non-experts, implying that an ARSG experience is related to enhancing the VWM capacity rather than the general fluid intelligence. This is consistent with previous findings which state that fluid intelligence is less plastic (Boot et al., 2008; Dale and Green, 2017a; Bavelier and Green, 2019; Sala and Gobet, 2019).

In addition, this study showed a greater VWM advantage at the larger set size for ARSG experts (see Figure 2; the advantage of accuracy: size 1 < size 2 < size 4). Past studies suggested that individual differences of VWM capacity may be more evident at larger sizes, which is associated with a greater requirement for attentional selectivity and control (Vogel et al., 2005; Fukuda and Vogel, 2009; Kuo et al., 2012; Blacker and Curby, 2013). Furthermore, research demonstrated that the underlying mechanism of the AVG players’ superior VWM may enhance selective attention by improving attentional control and executive functioning (Green and Bavelier, 2012). Thus, consistent with previous findings (Green and Bavelier, 2012; Blacker and Curby, 2013), the current finding that ARSG experts had a greater VWM advantage at the larger sizes suggested that improved attentional ability may devote to this VWM advantage. Furthermore, we found that, in the ARSG non-experts, the accuracy rate significantly decreased from size 1 to size 2 then to size 4, but in the ARSG experts, it did not differ between size 1 and size 2, which may be due to a ceiling effect (size 1 = 0.98; size 2 = 0.97). These results also demonstrated that ARSG experience is related to a significant enhancement in VWM performance.

Although our findings were consistent to: (1) previous studies that found ARSG or AVG experience to be related to the enhancement of attention (Green and Bavelier, 2003; Qiu et al., 2018; Gan et al., 2020); and (2) substantial evidences showing the close relation between VWM and attention (Vogel et al., 2005; Astle and Scerif, 2011; Kuo et al., 2012), the correlational nature of this study precludes drawing causal inferences about the adaptive effect of ARSG experience on the improvement of VWM. Future interventional research should systematically explore this developmental benefit of video gaming. Next, our findings demonstrated that a prolonged ARSG experience was related to the enhancement of the VWM capacity, and improved attentional ability may devote to this VWM enhancement; however, the potential role of other cognitive abilities in contributing to the ARSG players’ VWM advantage still requires further research.

## Conclusions

This study examined the behavioral and ERP data in ARSG experts and non-experts at a visual change detection task to determine whether a long-term ARSG experience was relative with an enhanced VWM capacity. The behavioral results showed that an ARSG experience was relative to an enhanced VWM performance, similar to the AVG genre. Furthermore, using CDA component (taken as a more precise masker for VWM capacity than behavioral performance) by ERP measure, the present study revealed the electrophysiological mechanism underlying the positive relationship between ARSG experience and enhancing the VWM capacity. Thus, our data indicated that a prolonged ARSG experience was related to improvements in the VWM capacity. However, because of the correlational nature of this study, the causal relationship between ARSG experiences on VWM capacity requires further research.

## Data Availability Statement

The raw data supporting the conclusions of this article will be made available by the authors, without undue reservation.

## Ethics Statement

The studies involving human participants were reviewed and approved by the ethics research committee of the University of Electronic Science and Technology of China (UESTC). The patients/participants provided their written informed consent to participate in this study.

## Author Contributions

RC, DG, and YY performed the study design, data collection, data analysis, result interpretation, and initial manuscript writing. WM, GY, and TL contributed to the study design, data analysis, result interpretation, and initial manuscript writing. YL, LZ, JJ, NQ, and LD conducted the data analysis, result interpretation, and initial manuscript writing. All authors contributed to the article and approved the submitted version.

## Conflict of Interest

The authors declare that the research was conducted in the absence of any commercial or financial relationships that could be construed as a potential conflict of interest.
